# The Dual Roles of MYC in Genomic Instability and Cancer Chemoresistance

**DOI:** 10.3390/genes8060158

**Published:** 2017-06-07

**Authors:** Alpana Kumari, Watson P. Folk, Daitoku Sakamuro

**Affiliations:** 1 Department of Biochemistry and Molecular Biology, Medical College of Georgia, Augusta University, Augusta, GA 30912, USA; fwatson1@augusta.edu (W.P.F.); dsakamuro@augusta.edu (D.S.); 2 Tumor Signaling and Angiogenesis Program, Georgia Cancer Center, Augusta University, Augusta, GA 30912, USA; 3 Biochemistry and Cancer Biology Program, The Graduate School, Augusta University, Augusta, GA 30912, USA

**Keywords:** MYC, genomic instability, chemoresistance, γH2AX

## Abstract

Cancer is associated with genomic instability and aging. Genomic instability stimulates tumorigenesis, whereas deregulation of oncogenes accelerates DNA replication and increases genomic instability. It is therefore reasonable to assume a positive feedback loop between genomic instability and oncogenic stress. Consistent with this premise, overexpression of the MYC transcription factor increases the phosphorylation of serine 139 in histone H2AX (member X of the core histone H2A family), which forms so-called γH2AX, the most widely recognized surrogate biomarker of double-stranded DNA breaks (DSBs). Paradoxically, oncogenic MYC can also promote the resistance of cancer cells to chemotherapeutic DNA-damaging agents such as cisplatin, clearly implying an antagonistic role of MYC in genomic instability. In this review, we summarize the underlying mechanisms of the conflicting functions of MYC in genomic instability and discuss when and how the oncoprotein exerts the contradictory roles in induction of DSBs and protection of cancer-cell genomes.

## 1. Introduction: MYC as a Transcription Factor

The MYC family of transcription factors directly and indirectly regulates various genes involved in cell proliferation [1], cellular metabolism [2], induction of apoptosis [3], blocking of differentiation [4], and initiation and promotion of cancer [5,6,7]. There are three MYC proto-oncoproteins, c-MYC, N-MYC, and L-MYC, which are encoded by separate genes and have different tissue distribution and oncogenic potency [8,9,10]. Deregulated expression of c-MYC is associated with the development of most human malignancies [11,12,13], whereas overproduction of N-MYC is frequently associated with neuroblastomas and gliomas [14,15]. In contrast, L-MYC expression is evident in both neonatal and adult lung tissues and most frequently overexpressed in lung cancer, including small-cell lung carcinomas [9], suggesting a non-redundant biology sustained by each member of the MYC family of transcription factors. However, in the absence of c-MYC, the majority of c-MYC-dependent gene transcription [16] and c-MYC-dependent tissue development [17] were compensated by the re-establishment of N-MYC, implying a functional redundancy as sequence-specific transcription factors due to the structural similarity of each MYC protein.

The MYC transcription factor contains the amino-terminal (N-terminal) transcriptional activation domain (TAD), followed by the central region, the nuclear localization motif, and the carboxyl-terminal (C-terminal) basic-helix-loop-helix-leucine zipper (bHLH-LZ) domain. The N-terminal TAD region harbors at least two conserved segments, termed MYC boxes 1 and 2 (MB1 and MB2). Depending on the availability of MB1- and/or MB2-interacting coactivators and corepressors, such as the transformation/transcription domain–associated protein (TRRAP) [18], the pocket protein p107 [19], and a pro-apoptotic corepressor bridging integrator 1 (BIN1) [20,21], the MYC TAD region is essential for fine-tuning MYC-dependent transcriptional activation and repression [10,22].

The C-terminal bHLH-LZ region is required for hetero-dimerization with MYC-associated factor X (MAX), which allows MYC to recognize the MYC-binding consensus sequence “CACGTG”, termed the Enhancer box (E-box). MYC–MAX heterodimer formation is crucial for MYC-dependent gene transcription, cell proliferation, apoptosis, and oncogenic transformation [23,24], but MYC may also function as a transcription factor in a MAX-independent manner [25,26,27,28], implying the presence of a non-MAX heterodimeric binding partner of MYC to recognize the E-box. Furthermore, MYC directly interacts with MYC-interacting Zinc finger protein 1 (MIZ1) [29], which recognizes an initiator (INR) element, PyPyAN[T/A]PyPy (Py: Pyrimidine; N: any nucleotide) [30], in a core promoter region. Through direct MYC–MIZ1 interaction via an INR sequence of several growth-arresting genes, MYC cancels MIZ1-dependent transactivation [29,31,32,33]. In addition to a new emerging research area regarding the transcription-independent function of MYC, particularly in protein translation and DNA replication [34], it is important to identify downstream effectors of MYC-dependent gene transcription to better understand the multiple roles of MYC in various cell behaviors, such as cell proliferation survival, apoptosis, genomic instability, and cancer.

## 2. MYC in Cancer Development and Cancer Stemness

The critical involvement of MYC in cell growth was evident from the study showing rapid and sustained induction of MYC, following mitogenic stimulation of quiescent cells [35,36]. Furthermore, homozygous inactivation of *Myc* in immortalized rat fibroblasts resulted in prolonged doubling time and accumulation of cells in the G_1_ and G_2_/M phases in the cell cycle [37]. MYC abundance is consistently elevated during embryogenesis and in highly proliferative tissues, such as skin epidermis and gut of adults [38]. The first evidence for MYC as an immortalizing factor came from a classical RAS co-transformation assay using primary rodent fibroblasts as an in vitro cancer model system [39]. A number of studies have since been conducted providing evidence that MYC overproduction was sufficient to induce tumorigenesis both in vitro and in vivo [40,41,42]. Accordingly, an elevated or deregulated level of MYC is a hallmark of the vast majority of human malignancies and is usually recognized as a poor prognostic marker, indicating a strong link between deregulation or overexpression of the *MYC* gene and advanced cancer development.

It is important that MYC has been identified as one of the “Yamanaka” factors, along with three other distinct transcription factors, SOX2, OCT4, and KLF4, which are all essential for the establishment of inducible pluripotent stem (iPS) cells by reprograming transcriptional networks of fully differentiated somatic cells [43,44,45]. The growth behaviors of iPS cells are normally sustained properly. However, presumably because of ectopically carrying the *MYC* allele, iPS cells share several cellular properties with cancer cells naturally overexpressing MYC, such as malfunctioned senescence and cellular immortality [46,47]. MYC-expressing iPS cells could be therefore oncogenically transformed once proper or strict biological control of MYC activity has disappeared. Intriguingly, recent findings demonstrated that oncogenic MYC preserves cancer stemness, which enables cancer cells to survive longer with increasing metastatic potential even under genotoxic conditions [6,48]. Given that MYC participates in transcriptional reprogramming that facilitates the creation of iPS cells from differentiated cells [43,44,45], it may be possible for MYC-overexpressing cancer cells to acquire traits similar to those of cancer-stem cells [49,50,51]. It is undoubtedly envisioned that the failure of stringent regulation of MYC activity is associated with aberrant growth behaviors leading to the development of cancer stemness.

## 3. Oncogenic Addiction to MYC as an “Achilles’ Heel” of MYC-Driven Cancer Cells

Oncogene addiction represents a dependency of cancer cells on a single oncogenic protein (or pathway) for maintaining their survival and malignant properties [52,53]. Because of the strong correlation between MYC overexpression and tumorigenesis, it is anticipated that survival of cancer cells would be greatly compromised if MYC is inactivated. The depletion of MYC by the co-transfection of antisense RNA of c-MYC in cell lines derived from human tumors shows inhibition of proliferation and induction of differentiation [54,55], serving as the first experimental evidence of MYC addiction in cancer cells in vitro. Using in vivo model systems with conditional transgenic mice, inactivation of *MYC* consistently resulted in tumor regression in vivo in hematopoietic and solid tumors [56,57]. Of note, brief inactivation of MYC is sufficient to induce long-term loss of neoplastic phenotypes [58], indicating that oncogenic addiction elicited by MYC serves as an “Achilles’ heel” of MYC-expressing cancers [53,59]. However, because of the limited availability of a MYC-specific small-molecule inhibitor with little or no adverse effect in clinical settings [60,61], a strategy for therapeutically targeting MYC addiction seems fascinating, but remains frustratingly difficult.

## 4. MYC-Induced Replication Stress, Genomic Instability, and Oncogenic Transformation

During cell division, chromosomal DNA is entirely replicated, but errors in this process may result in various forms of genetic alterations, such as point mutations and gene amplifications [62]. A process prone to genomic alterations is termed as genomic instability, which could be one of the major causes of cancer initiation [63]. Chromosomal alteration, a genomic instability at the chromosomal level, has long been recognized to be the hallmark of human cancer [11,64]. It is well acknowledged that a tumor cell is the progeny of a single genetically unstable cell, which continuously acquires chromosomal abnormalities over time [65]. The genomic instability driving oncogenesis can be further fueled by errors in DNA replication. Some cells may accumulate more genetic changes in their chromosomes, which subsequently provide selective advantages for growth [66,67]. The majority of cancers exhibit deregulated expression of MYC as a result of either activation of upstream regulatory signals, such as WNT-dependent *MYC* expression [68] and Src-dependent MYC activation [69], or chromosomal instability, which causes gene amplification, insertional mutagenesis, and gene translocation [70,71,72]. For example, gene amplification of the c-*MYC* locus is one of the most representative gene abnormalities in prostate cancer [73].

In contrast, deregulated MYC could be a trigger of chromosomal instability presumably as a consequence of MYC-induced aberrant DNA replication and cell division [74]. Deregulated MYC expression may result in uncontrolled activation of a number of downstream genes, which promote the cell cycle and DNA synthesis, and ultimately initiates and promotes genomic instability [75]. In agreement with this premise, MYC induction is often associated with a variety of chromosomal changes, for example, generation of extrachromosomal elements [76], centromere and telomere fusions [77], chromosome and chromatid breaks, translocations, deletions, inversions, and aneuploidy [74,75,77,78,79].

The close relationship between MYC and chromosomal instability was further proven by the studies showing MYC-mediated induction of nonrandom amplification and rearrangement of the genes encoding several growth-promoting genes, such as dihydrofolate reductase (DHFR), carbamyl-P synthetase, aspartate transcarbamylase, dihydro-orotase, cyclin D2, and the R2 subunit of ribinucleotide reductase [80,81,82,83,84]. Interestingly, human pre-invasive cervical cancer shows *DHFR* amplification in association with MYC overexpression [85]. Unless apoptosis or cellular senescence is activated, MYC-induced chromosomal rearrangements would provide a proliferative advantage to MYC-overexpressing cells, which may subsequently induce even more genomic instability, including double stranded DNA breaks (DSBs). Consistent with this idea, deregulated MYC upregulates the formation of γH2AX foci, a surrogate biomarker of DSBs [86,87]. In normal human foreskin fibroblasts, MYC overexpression is sufficient to increase the formation of γH2AX foci [88].

A possible mechanism by which MYC induces DSBs is through accumulation of reactive oxygen species (ROS) [89,90]. However, MYC-induced DSBs also occur in a manner independent of ROS production, implying that a different mechanism may also contribute to MYC-mediated production of DSBs [91]. For example, several studies support the possibility that overexpression of MYC represses cellular DSB repair potentials [88,92,93]. It is important that replication stress causes aberrant replication intermediates and MYC is actively involved in DNA replication, particularly initiation of replication [94,95]. Overexpressed MYC can function as an illegitimate replication-licensing factor [75]. MYC is also capable of overriding cell-cycle checkpoints, thereby triggering genomic instability during DNA replication [74]. Deregulated MYC may therefore induce DSBs by multiple mechanisms, such as accumulation of ROS, an increase in replication stress [96], and decrease in DSB repair capacity [88,92,93]. The tumorigenic nature of MYC is thus attributable to not only its pivotal roles in the upregulation of genes promoting cell proliferation, but also its ability to promote genomic instability through chromosomal aberrations and DSBs.

In normal fibroblasts, oncogenic MYC-induced replication stress provokes genomic instability, which then stimulates cellular checkpoint mechanisms, including cell-cycle arrest, apoptosis, and premature senescence [97,98]. Hence, oncogene-induced checkpoints are thought to be cellular defense mechanisms that halt or delay the onset of tumorigenesis as long as the levels of DNA damage are not devastating [96]. Because oncogenic RAS [98,99] and oncogenic RAF , which functions downstream of the RAS protein family [100], can also induce premature senescence in normal fibroblasts, oncogene-induced checkpoint mechanisms are not MYC-specific functions, but broadly triggered by oncogenic stress and subsequent acceleration of cell-cycle progression and DNA hyper-replication [97,98]. Because tumorigenesis is promoted by genomic instability [96], any malfunction of the checkpoint machinery could be a potential cause of cellular transformation.

Using rodent primary embryo fibroblasts as an in vitro model system, oncogenic foci formation can be experimentally achieved by the co-transfection of two independent oncogenes; one is a nuclear oncogene (such as c-MYC and adenovirus E1A) and the other is a cytoplasmic oncogene (such as activated RAS) [39,101]. Since then, the requirement of concurrent activation of multiple oncogenes for cellular transformation has been experimentally established [102], which implies a functional collaboration between two independent oncogenic pathways to circumvent or inactivate cellular checkpoint mechanisms. BIN1 was identified as one of the 17 cellular anticancer proteins that promote apoptosis and senescence in response to oncogenic RAF [100]. Intriguingly, overexpression of c-MYC broadly represses the *BIN1* transcription in a manner dependent on MIZ1 [33]. It will be interesting to investigate whether oncogenic MYC represses or inactivates any other checkpoint-related cellular factors activated by oncogenic RAS and RAF.

## 5. MYC in DNA Repair and Cancer Chemoresistance

Current cancer treatments rely greatly on conventional chemotherapeutic drugs. Most of the clinically approved chemotherapeutic agents cause cytotoxicity in rapidly proliferating cells, such as cancer cells and hair-follicle cells, by inducing DNA damage. However, tumor cells often become refractory to a chemotherapeutic agent, a phenomenon known as chemoresistance. Cancer cells may have intrinsic drug-resistance or acquire it during genotoxic treatment [103]. The mechanisms by which advanced cancer cells increase chemoresistance could be attributable to (a) an increase in the amount (and/or activity) of cellular DNA repair machinery [104,105]; (b) suppression or inactivation of pro-apoptotic machinery [106,107]; and/or (c) detoxification and enhanced efflux of genotoxic chemicals [108,109]. Clinical evidence shows that once cancer cells acquire resistance to first-line chemotherapeutic drugs such as cisplatin, cancer cells naturally develop cross-resistance to a range of other chemotherapeutic agents, ultimately leading to treatment failure in over 90% of patients with metastatic diseases [108,110]. The major clinical challenge for effective chemotherapy is therefore the rapid elimination of chemosensitive cancer cells before chemoresistance appears.

In general, accumulation of genomic instability renders the cancer-cell genome vulnerable to DNA-damaging agents [111,112]. MYC-induced genomic instability, which increases cancer incidence (see above), might thus serve as a double-edged sword to eradicate cancer cells overexpressing MYC in combination with therapeutic DNA-damaging agents. Paradoxically, it is well known that the abundance of MYC oncoprotein closely correlates with chemoresistance in many tumor types, including prostate cancer, ovarian cancer, melanoma, lung cancer, and hepatocellular carcinoma both in vitro and in vivo [33,113,114,115,116]. In patients with ovarian cancer, high levels of MYC are coupled to tumor recurrence, poor overall survival, and cisplatin resistance [117].

Moreover, c-MYC expression is elevated in tumor-cell fractions that have survived platinum-based chemotherapy in vivo [113]. In embryonal rhabdomyosarcoma cell lines, MYC protects cancer cells from radiation-induced apoptosis and DNA damage, while promoting radiation-induced DNA repair [118]. In light of these reports, oncogenic MYC seems to be an attractive target to overcome chemoresistance in a wide range of human cancers. Consistent with this premise, antisense oligodeoxynucleotides or the silencing of MYC by small-interfering RNA increases cisplatin sensitivity in inherently cisplatin-resistant melanoma cell lines in vitro and reduces tumor formation in xenograft models of cisplatin-resistant ovarian cancer cells [117,119]. Moreover, the small-molecule MYC inhibitor, 10058-F4, was reported to be effective in anti-tumor treatment, such as for leukemia [120], prostate cancer [33], and hepatocellular carcinoma [116]. These in vitro and in vivo observations unfailingly suggest that oncogenic MYC protects cancer-cell genomes from therapeutic DNA-damaging agents.

As we discussed above, there is solid evidence that oncogenic MYC increases replication stress and genomic instability, which then initiates an early step leading to oncogenic transformation if cellular checkpoint mechanisms are inactivated. However, the maintenance of genomic integrity during DNA replication is one of the most fundamental biological traits for the preservation of genetic information, even though MYC, a powerful inducer of DSBs, always promotes the G_1_/S phase transition in the cell cycle. Notably, in addition to MYC, the E2F1 transcription factor is another preeminent transcription factor, single transfection of which is sufficient to promote G_1_/S transition in quiescent (i.e., serum-starved) fibroblasts [121]. Because E2F1 is well known to actively promote DSB repair via physically interacting with Nijmegen Breakage Syndrome Protein 1 (NBS1), a key component of the MRN (MRE11/RAD50/NBS1) DNA-end-binding complex [122,123,124], and transcriptionally stimulates the human c-*myc* gene promoter [125,126], there may be a possible collaboration between these two master cell-cycle-promoting transcription factors not only in the G_1_/S phase transition [121], but also in the synchronized activation of DSB-repair machinery. Hence, it is logical to hypothesize that similar to E2F1, MYC facilitates a DSB repair-promoting function to counterbalance (or, at least, mitigate) an unavoidable effect of MYC on replication stress-induced genomic instability during DNA replication.

Consistent with this premise, MYC physically associates with the 5′-flanking promoter region of various DSB repair-related genes, such as *NBS1*, *Ku70*, *Rad51*, *BRCA2*, *Rad50*, and the DNA-dependent protein kinase catalytic subunit (DNA-PKcs), and activates their transcription [127,128,129,130]. Possible involvement of MYC in DNA repair is evident, in addition, from the finding that the silencing of MYC was correlated with a decrease in kinase activities of ataxia-telangiectasia mutated serine/threonine protein kinase (ATM) and DNA-PKCs, which consequently resulted in a reduction in irradiation-induced DSB repair efficiency [131]. Consistently, there was a significant reduction in the capability of DSB repair in HeLa cells after the silencing of MYC [131]. It is pertinent to experimentally establish the effect of the impairment of endogenous MYC on DSB repair pathway(s) in various cultured cancer model systems. Interestingly, cancer stem cells are well known to survive longer, even under genotoxic circumstances, such as chemo and radiation therapy [132,133,134], and robustly upregulate cellular DNA-repair activity [135,136]. Because oncogenic MYC transcriptionally stimulates a number of DSB repair genes [127,128,129,130] and is responsible for the maintenance of cancer stemness [137,138], we propose that, under devastating genotoxic stress and/or during the maintenance of cancer stemness, oncogenic MYC protects the cancer-cell genome from DNA damage, thereby allowing advanced cancer (or cancer stem) cells to survive longer.

If so, inhibiting DNA repair following treatment with DNA-damaging agents could be a promising approach for eradicating MYC-induced chemoresistance. Small-molecule inhibitors that target DNA repair proteins, such as poly (ADP-ribose) polymerase (PARP) [139,140], DNA-PKCs [141,142], and ATM [143,144], are emerging as attractive targets for chemotherapy [145]. PARP inhibitors have been most widely tested as an anticancer therapeutic option [146]. Our group has previously shown that BIN1, originally identified as a MYC-interacting tumor suppressor [21], functions as a cellular inhibitor of PARP1 in vitro and that MYC induces cisplatin resistance by liberating intrinsic PARP1 activity through directly suppressing BIN1 levels [33]. We have also recently shown that PARP inhibition increases BIN1 levels, which play an important role in mediating E2F1-induced apoptosis, particularly under serum-starved conditions [147]. Because reduced blood supply is frequently observed in solid tumor tissues, especially following chemotherapy [148,149], induction of endogenous BIN1 expression by PARP inhibitors, such as olaparib, may be combined with conventional chemotherapy to reverse MYC-dependent chemoresistance.

## 6. Conclusions

Given that rapidly proliferating cancer cells, which naturally produce oncogenic MYC, accumulate more genomic instability, it is logical to assume that late-stage or recurrent cancer cells, which are expected to carry high levels of MYC, must have accumulated more DNA damage. Advanced (i.e., late-stage) cancer cells should subsequently be more susceptible to DNA-damaging chemotherapeutic drugs. However, the opposite occurs; late-stage cancer cells survive longer even during/after DNA-damaging chemotherapy [113,119]. This evidence clearly suggests that, in advanced cancer cells, oncogenic MYC develops a highly efficient DNA repair system and offsets the genomic instability induced by chemo and radiation therapy [118].

To better understand the functional discrepancy of the formation of γH2AX foci by oncogenic MYC in chemoresistant cancer cells, we must remember that most of the evidence that supports the MYC-induced DSBs in cancer cells mainly derives from the efficient formation of γH2AX foci by experimentally overexpressed MYC. Although γH2AX is generally considered to be the most reliable biomarker of DSBs, there are several reports showing DSB-independent functions of γH2AX [150,151,152]. ATM kinase is indeed responsible for the formation of γH2AX in response to DSBs, but the same enzyme can also be activated in a DSB-independent manner [153], such as hypoxia [154], where γH2AX foci can be formed in a manner independent of DSBs.

**Figure 1:**
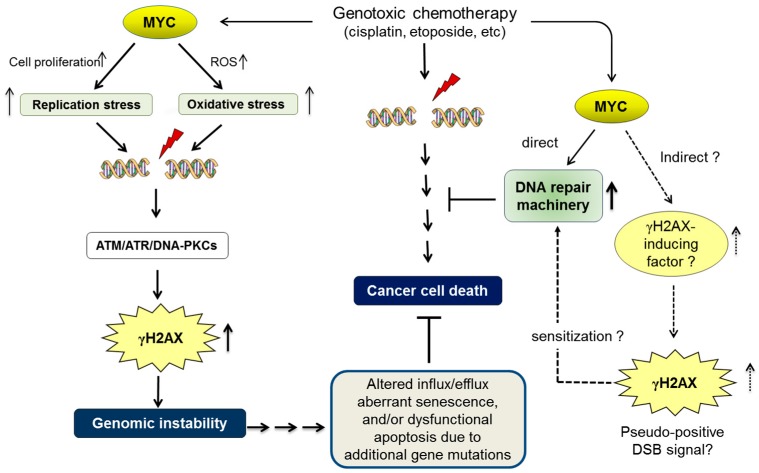
MYC induces genomic instability and chemoresistance.

If MYC increases γH2AX foci in a DSB-independent manner, one possible model is to stimulate a γH2AX-inducing factor independent of DSBs. Consistent with this idea, it has been reported that MYC is required for the activation of ATM and formation of γH2AX foci [155], whereas suppression of ATM dramatically sensitizes tumors to DNA-damaging chemotherapy [156]. We propose that upregulation of the γH2AX-inducing factor (such as ATM) by MYC in cancer cells might result in persistent formation of γH2AX foci, which serves as a pseudo-positive DSB signal that sustains the activation of DNA-repair machinery (Figure 1).

Deregulated MYC induces replication stress and oxidative stress due to reactive oxygen species (ROS), which subsequently contribute to promoting double-stranded DNA breaks (DSBs). DSBs upregulate the phosphorylation of histone H2AX (forming γH2AX) by activating the sensors of DNA damage, such as ataxia-telangiectasia mutated serine/threonine protein kinase (ATM), ATM- and Rad3-related protein kinase (ATR), and/or DNA-dependent protein kinase (DNA-PK). γH2AX is widely recognized as a surrogate biomarker of DSBs. A MYC-dependent increase in γH2AX foci may therefore be indirect evidence of increased genomic instability by MYC. Genomic instability is a consequence of oncogenic MYC, but it may also act as an upstream factor that further promotes malignant phenotypes. For example, additional gene mutations due to genomic instability may have negative influence on cancer-cell death machinery by altering the influx/efflux, cellular senescence, and apoptotic/survival signals [106,107,108,109]. Although genotoxic chemotherapeutic drugs effectively kill cancer cells by inducing deleterious DSBs, it is widely recognized that high levels of MYC eventually render cancer cells resistant to genotoxic drugs [117]. In addition to the direct upregulation of DNA-repair machinery [130], we propose that deregulated MYC induces chemoresistance indirectly by activating a γH2AX-inducing factor, such as ATM [131,155], which subsequently increases γH2AX foci as a pseudo-positive DSB signal and sensitizes the cellular DNA-repair machinery.

However, it is our consensus that the underlying mechanisms of cancer chemosensitivity depend, at least in part, on intrinsic tumour suppressor mechanisms, such as TP53 [107] and BIN1 [33], by which DSB-induced apoptosis or senescence can be elicited. Moreover, cancer chemoresistance relies, to some extent, on enhanced efflux and/or detoxification of anticancer drugs [109,157]. Therefore, in addition to the development of DSB-repair function by MYC in late-stage cancer cells, deregulated MYC, which leads to increased genomic instability, at least partly, via replication stress (see above), offers a chance for cancer cells to accumulate more genetic mutation and develop ways of evading the cytotoxic effects of anticancer drugs by, for example, downregulating tumour suppressor functions and/or upregulating drug efflux and detoxification (Figure 1).

In this review, we have discussed the dual roles of MYC in the development of genomic instability and chemoresistance in cancer cells. Further work, such as identification of downstream MYC effectors that liberate ATM-dependent formation of γH2AX foci, is important to reconcile the contradictory nature of this enigmatic oncoprotein in response to DNA damage.
